# Supplementary material for: Predicting visual function by interpreting a neuronal wiring diagram
Source: Nature. 2024 Oct 2;634(8032):113–23. doi: 10.1038/s41586-024-07953-5 (PMC11446822; doi:10.1038/s41586-024-07953-5)
Supplement: Supplementary file 6 — CRF and ERF predictions for individual TmY4 and TmY9 cells. Analogous to Supplementary Data 3, but for TmY target types. Shown are the top four monosynaptic pathways, the strongest pathway passing through each of the top ten intermediary types (ranking from Extended Data Fig. 7), and the trisynaptic pathway Tm1–TmY–Dm3–TmY (see the section entitled Prediction of spatial normalization). [file 41586_2024_7953_MOESM6_ESM.zip › DataS4/TmY9q/720575940627222440.pdf]

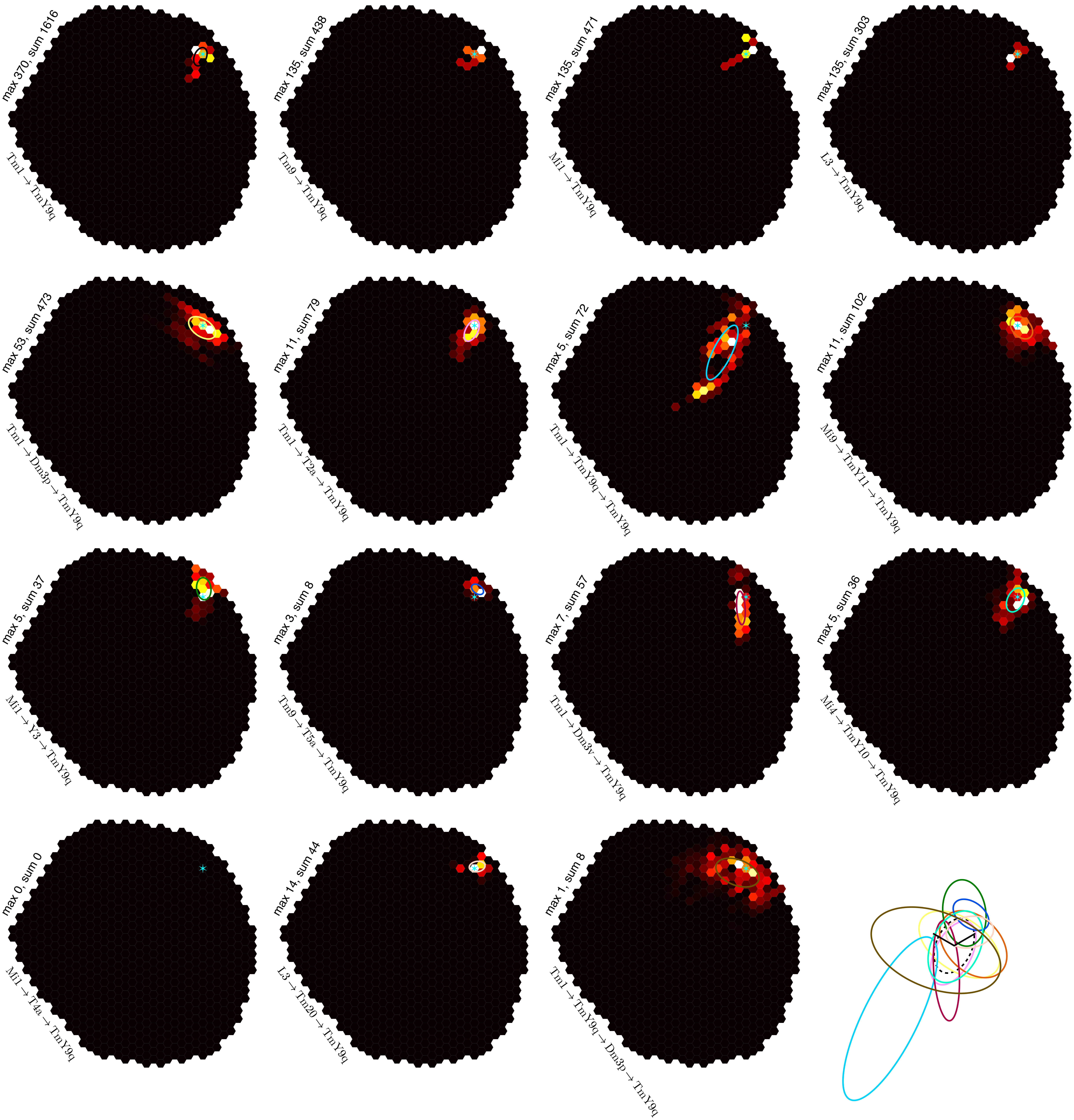
max 370, sum 1616
Tm1 → TmY9q
max 135, sum 438
Tm9 → TmY9q
max 135, sum 471
Mi1 → TmY9q
max 135, sum 303
L3 → TmY9q
max 53, sum 473
Tm1 → Dm3p → TmY9q
max 11, sum 79
Tm1 → T2a → TmY9q
max 5, sum 72
Tm1 → TmY9q → TmY9q
max 11, sum 102
Mi9 → TmY11 → TmY9q
max 5, sum 37
Mi1 → Y3 → TmY9q
max 3, sum 8
Tm9 → T5a → TmY9q
max 7, sum 57
Tm1 → Dm3v → TmY9q
max 5, sum 36
Mi4 → TmY10 → TmY9q
max 0, sum 0
Mi1 → T4a → TmY9q
max 14, sum 44
L3 → Tm20 → TmY9q
max 1, sum 8
Tm1 → TmY9q → Dm3p → TmY9q
